# Prognostication after out-of-hospital cardiac arrest, a clinical survey

**DOI:** 10.1186/1757-7241-16-9

**Published:** 2008-09-15

**Authors:** Michael Busch, Eldar Søreide

**Affiliations:** 1 Dept. of Anaesthesiology and Intensive Care, Stavanger University Hospital, Postboks 8100, 4068 Stavanger, Norway

## Abstract

**Background:**

Numerous parameters and tests have been proposed for outcome prediction in comatose out-of-hospital cardiac arrest survivors. We conducted a survey of clinical practice of prognostication after therapeutic hypothermia (TH) became common practice in Norway.

**Methods:**

By telephone, we interviewed the consultants who were in charge of the 25 ICUs admitting cardiac patients using 6 structured questions regarding timing, tests used and medical specialties involved in prognostication, as well as the clinical importance of the different parameters used and the application of TH in these patients.

**Results:**

Prognostication was conducted within 24–48 hours in the majority (72%) of the participating ICUs.

The most commonly applied parameters and tests were a clinical neurological examination (100%), prehospital data (76%), CCT (56%) and EEG (52%). The parameters and tests considered to be of greatest importance for accurate prognostication were prehospital data (56%), neurological examination (52%), and EEG (20%).

In 76% of the ICUs, a multidisciplinary approach to prognostication was applied, but only one ICU used a standardised protocol. Therapeutic hypothermia was in routine use in 80% of the surveyed ICUs.

**Conclusion:**

Despite the routine use of TH, outcome prediction was performed early and was mainly based on prehospital information, neurological examination and CCT and EEG evaluation. Somatosensory evoked potentials appear to be underused and underrated, while the importance of prehospital data, CCT and EEG to appear to be overrated as methods for making accurate predictions.

More evidence-based protocols for prognostication in cardiac arrest survivors, as well as additional studies on the effect of TH on known prognostic parameters are needed.

## Introduction

It has been estimated that approximately 275000 Europeans experience out-of-hospital cardiac arrest (OHCA) every year [1]. When cardiopulmonary resuscitation (CPR) attempts are made, a return of spontaneous circulation (ROSC) may be achieved in up to half of the victims, leading to an estimated number of up to 116000 hospital admissions annually in Europe [2]. Almost 80% of patients who initially survive an OHCA remain in a coma for varying lengths of time and are admitted to an ICU [3]. About two-thirds of these patients die during the subsequent hospital stay: the majority dies due to neurological injury [4]. After implementation of post-resuscitation therapeutic hypothermia (TH), these numbers have improved dramatically [5,6]. Still, early and reliable prognostication of neurological outcome is essential to prevent futile treatment, ease the emotional burden on family members and ensure cost-effective resource management.

Several studies, reviews and specialised groups have attempted to devise improved criteria for cerebral prognostication in OHCA arrest victims. In 2002, members of the Austrian interdisciplinary consensus conference identified 26 parameters with varying evidence-levels, that allowed the clinician to make a prognostic assertion [7]. A more recent systematic review by Wijdicks et al. suggests a decision algorithm for use in prognostication, that includes brain stem reflexes, motor response, myoclonus status epilepticus, somatosensory evoked potentials (SSEP) and serum neuron specific enolase (NSE) [8].

Neither the European Resuscitation Council (ERC) nor the American Heart Association (AHA) guidelines offer a protocol-type approach to prognostication [9,10]. Our aim was to study current clinical practice of post-resuscitation prognostication in Norway after nation-wide implementation of TH.

## Methods

In May 2005, we conducted a semi-structured telephone survey of all Intensive Care Units (ICUs) of the Norwegian Intensive Care Registry (NIR). The consultant responsible for the ICU answered 6 structured questions, including the time the cerebral prognostication was made, the medical specialties involved, the specific prognostic tests applied, and a personal assessment with regard to the clinical importance of the different tests and parameters (see Additional file 1). Furthermore, we documented the use of a standardised prognostication protocol and therapeutic hypothermia. The respondents could choose several alternatives, so that the sum of responses in percentages may add up to more than 100%. Results are presented as descriptive statistics (Microsoft Office Excel).

## Results

**Figure 1:**
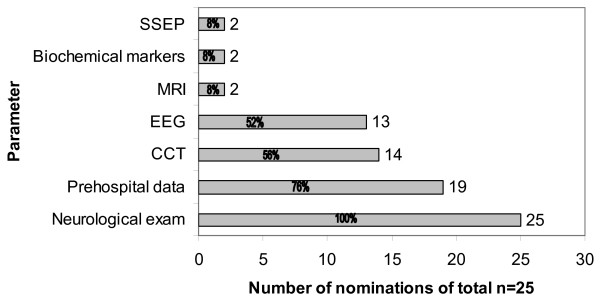
**Applied prognostic parameters.** Figure 1 depicts the frequency of application of different prognostic parameters.

Twenty-five of the 27 ICUs (92%) reporting data to the Norwegian Intensive Care Registry (NIR) participated in the survey. All geographic regions of Norway were represented. The two non-participating hospitals are located in different parts of Norway, and their importance is negligible with regard to the numbers of post-resuscitation care patients. The participating ICUs caring for comatose OHCA survivors were predominately led by anaesthesiologists (96%). However, a multidisciplinary approach to prognostication was applied in 19 hospitals (76%), with anaesthesiology, cardiology and neurology being the specialties involved. In the remaining six institutions (24%), anaesthesiology (n = 2), cardiology (n = 1) and neurology (n = 3) were sole responsible for outcome prediction. In 18 ICUs (72%), prognostication was conducted within 24–48 hours after hospital admission. In the remaining ICUs, it was conducted within 48–72 hours. The primary methods used to predict outcome were clinical neurological examination and prehospital data (figure 1). SSEP played a minor role (8%) 2/25). With regard to prognostication based on prehospital data, the elements listed most were: initial ECG rhythm (100%), witnessed arrest (84%), bystander cardiopulmonary resuscitation (CPR) (53%), prior health status (47%), presumed no-flow time (21%), and CPR duration to return of spontaneous circulation (ROSC) (21%).

The respondents rated prehospital data (56%), neurological examination (52%), and EEG (20%) to be of greatest prognostic importance, with the corresponding number for SSEP being (8%), biochemical markers (4%), CCT (4%) and MRI (4%).

Eighty percent (n = 20) of the ICUs used post resuscitation TH routinely. Only one ICU used a standardised approach to predict outcome.

## Discussion

A multitude of parameters, models and tests have been proposed for post- resuscitation prediction of cerebral outcome in comatose OHCA survivors [3,7-12]. The strength and level of evidence of current predictors vary widely. With TH now becoming the standard of care for this type of injury, the currently used prognostic parameters may need revision [8,13]. At the time of our study, 80% of Norwegian ICUs already used TH routinely. Three quarters of the ICUs used a multidisciplinary approach to increase diagnostic accuracy, but only one institution applied a standardised protocol. We think our results mirror the complexity of prognostication in anoxic-ischemic coma and underline the need for international evidence- based guidelines.

There is a general international consensus that prognostication should be delayed until day 3 after cardiac arrest [9,10,12]. By day 3, approximately half of those patients with ultimately poor prognosis have died, and clinical neurological examination enables the clinician to select about 50% of those remaining in this poor prognosis group [14]. Comas persisting beyond 3 days carry a greater than 90% risk of poor outcome [11]. In our study, 100% of prognostication was performed within 72 hours of hospital admission after cardiac arrest, and more surprisingly, 74% (n = 17) were conducted within 24–48 hours. Even though the time of prognostication does not necessarily need to coincide with the decision to withdraw active treatment, we think there is a substantial risk that negative expectations during the first days of treatment may affect patient management and subsequent outcome.

Neurological examination was the most frequently used prognostic parameter in our study. The current ERC guidelines state that no neurological sign is able to predict outcome in the first hours after return of spontaneous circulation (ROSC), but the absence of pupil light reflexes on day 3 and an absence of motor response to pain on day 3 are both independent predictors of poor outcome [9]. A systematic review by Wijdicks and colleagues identified an absent or extensor motor response after 3 days, absence of pupillary or corneal reflexes within 1–3 days after CPR and myoclonus status epilepticus within the first 24 hours as clinical findings with a false predictive rate (FPR) of zero with narrow confidence intervals (CIs) for patients with invariably poor neurological prognosis [8]. Thus, in the absence of confounding factors, the clinical neurological examination may represent a reliable method to predict outcome.

Circumstances surrounding the OHCA were the second most frequent prognostic parameter used in our study and were also rated to be the parameter with the greatest clinical importance. Several parameters have been shown to be independent predictors of poor outcome: age over 70 years, co-morbidities, no-flow time, duration of cardiopulmonary resuscitation (CPR), and the cause of cardiac arrest and initial rhythm [7,15-17]. No-flow time, duration of CPR, initial rhythm and cause of cardiac arrest, however, cannot discriminate accurately between poor and favourable outcome with false predictive rates (FPR) ranging from 20–27% with narrow confidence intervals (CIs) [8]. Therefore, the current international resuscitation guidelines do not refer to prehospital data as a predictive parameter for prognostication in anoxic coma [9,10]. Our survey indicates that clinicians overrate the predictive value of such prehospital data.

With regard to neuroimaging, cerebral computer tomography (CCT) scan was the third most frequently applied prognostic parameter in our survey. Still, it was rated as being of minor importance in prognostication. In the literature, CCT is suggested only to exclude primary cerebral causes of the cardiac arrest and coma, as there is insufficient evidence that CCT-findings could conclusively prognosticate poor outcome in anoxic coma [8,12]. Several studies have associated pathological magnetic resonance imaging (MRI) signal changes with poor neurological prognosis [18,19]. Conventional MRI and diffusion-weighted MRI are superior in depicting pathophysiological alterations after global cerebral hypoxia in the cortex, cerebellum and basal ganglia when compared with conventional CCT [7]. In our study, MRI played only a minor role in prognostication, which may be due to its unknown predictive value for poor outcome and/or the safety concerns related to transport of a critically ill patient to the MRI lab [8,20].

Electrophysiological tests in coma prediction consist of evoked potentials (EP) and electroencephalogram (EEG). According to the recent ERC guidelines on resuscitation, an EEG performed at least 24–48 hours after cardiac arrest provides only limited prognostic information [9]. A normal or grossly abnormal EEG may predict outcome accurately, but an EEG between these values is unreliable for prognostication [7,12,21]. In spite of the insufficient predictive value of EEG and the substantial susceptibility to other factors such as drugs, sepsis and electrolyte disturbances, the respondents in our survey frequently used EEG and rated it to be of major clinical importance.

Somatosensory evoked potentials (SSEP) are much less influenced by drugs, metabolic derangements or therapeutic hypothermia [22,23]. Systematic reviews of outcome prediction and the current international guidelines on resuscitation have concluded that SSEP accurately predict a poor outcome, when bilateral absence of N20 is recorded 1–3 days after CPR [8-10,12]. Surprisingly, we found that SSEP were neither applied routinely nor deemed to be of clinical importance. This may be partially explained by the limited availability, and therefore lack of experience with this technique.

We found that biochemical markers were rarely used to predict outcome. This concurs with the recent international resuscitation guidelines and systematic reviews that state that measurement of biochemical markers theoretically may be useful, but that the results lack sufficient predictive accuracy [8-10].

A survey such as ours does have some limitations. Only one clinician was interviewed at each institution, and it cannot be guaranteed that the data represent the general practice at the hospital, even though the interviewed physicians were all on a consultant level with direct responsibility for the ICU. Despite the small number of surveyed hospitals, our study represents almost all the ICUs in Norway providing post resuscitation care. Our findings, however, have limited validity outside of Norway. A structural weakness of the survey was that the time of cerebral prognostication was defined as the time the consult was made, but that no information was gathered as to whether the results led to changes or withdrawal of active treatment.

Nontheless, we think our national survey represents an important insight into current clinical practice in an era of increased focus on the post-resuscitation phase in addition to new treatment modalities such as therapeutic hypothermia as well as a major focus on timely and accurate prognostication [8,9,24,25].

## Conclusion

Most Norwegian ICUs providing post-resuscitation care in comatose survivors of OHCA make use of TH. Despite the fact that this may complicate early neurological outcome prediction, the prognostication was performed early in the ICU phase (24–72 hours). The outcome prediction was mainly based on prehospital information, clinical neurological examination, CCT, and EEG evaluation. SSEP seemed to be underused and underrated, while the importance of prehospital data, CCT and EEG appeared to be overrated as predictors. We think our findings highlight the importance of establishing international evidence-based protocols for follow-up and prognostication in comatose OHCA survivors. Additionally, more studies on the effect of TH on known prognostic parameters are required.

## Competing interests

The authors declare that they have no competing interests.

## Authors' contributions

MB and ED participated in the design and coordination of the study. MB carried out the telephone survey and documented the data. MB and ED wrote the manuscript and performed the statistical analysis. All authors read and approved the final manuscript.

## Supplementary Material

Additional file 1 Cerebral prognostication after out-of-hospital cardiac arrest- questionnaire. The document depicts the questionnaire with which the survey was conducted. Click here for file
