# Supplementary material for: Prognostication after out-of-hospital cardiac arrest, a clinical survey
Source: Scand J Trauma Resusc Emerg Med. 2008 Sep 15;16:9. doi: 10.1186/1757-7241-16-9 (PMC2568950; doi:10.1186/1757-7241-16-9)
Supplement: Additional file 1 — Cerebral prognostication after out-of-hospital cardiac arrest- questionnaire. The document depicts the questionnaire with which the survey was conducted. [file 1757-7241-16-9-S1.doc]

Cerebral prognostication after out-of-hospital cardiac arrest

1. **At what time after the return of spontaneous circulation (ROSC) is cerebral prognostication performed ?**

⁯ first 24 hours

⁯ 24-48 hours

⁯ 48-72 hours

⁯ after 3 days

⁯ after 1 week

1. **Which medical specialties are involved in the prognostication of cardiac arrest survivors?**

⁯ Anaesthesiology

⁯ Internal Medicine

⁯ Neurology

⁯ other ____________

⁯ Multidisciplinary

1. **Which specific methods are applied for prognostication?**

⁯ Prehospital data (witnessed arrest, bystander CPR, initial ECG rhythm, no-flow time, CPR duration, prior health status)

⁯ Neurological examination

⁯ Somatic somatosensory evoked potentials (SSEP)

⁯ Electroencephalogram (EEG)

⁯ Biochemical markers

⁯ Cerebral computer tomography (CCT)

⁯ Magnetic resonance imaging (MRI)

⁯ Other____________________

1. **According to you, which of the prognostic methods mentioned above has the greatest sensitivity/specificity?**

⁯ Prehospital data

⁯ Neurological examination

⁯ Somatic somatosensory evoked potentials (SSEP)

⁯ Electroencephalogram (EEG)

⁯ Biochemical markers

⁯ Cerebral computer tomography (CCT)

⁯ Magnetic resonance imaging (MRI)

⁯ Other____________________

1. **Do you use a standardised protocol for cerebral prognostication after OHCA?**

⁯ Yes ⁯ No

1. **Do you use therapeutic hypothermia after OHCA in your ICU?**

⁯ Yes ⁯ No
